# The Effect of Vitamin D on Inflammation and Dyslipidemia in Type 2 Diabetes Mellitus: Protocol for a Systematic Review and Meta-analysis of Randomized Controlled Trials

**DOI:** 10.2196/42193

**Published:** 2023-03-14

**Authors:** Rizqah MacGirlley, Kabelo Mokgalaboni

**Affiliations:** 1 Department of Life and Consumer Sciences College of Agriculture and Environmental Sciences University of South Africa Roodepoort South Africa

**Keywords:** vitamin D, dyslipidemia, inflammation, hyperglycemia, type 2 diabetes mellitus, systematic review, chronic disease, mortality

## Abstract

**Background:**

Type 2 diabetes mellitus is a chronic disease that contributes to an increasing global burden on the health system. It has a high chance of leading to macrovascular complications and cardiovascular disease. As an inflammatory condition, it would be essential to target inflammatory pathways when developing therapeutic drugs for type 2 diabetes mellitus.

**Objective:**

We aimed to evaluate the effect of vitamin D on markers of inflammation and lipid profile among adult patients with diabetes.

**Methods:**

A systematic review will seek studies published on Embase, Google Scholar, PubMed, Web of Science, and Science Direct. This planned systematic review and meta-analysis will be limited to randomized controlled trials; moreover, the search will include published studies regarding the effects of vitamin D on pro-inflammatory cytokines and lipid profiles. The review will include studies published from inception until December 30, 2022. The study identification and selection will be based on the eligibility criteria by 2 independent reviewers. Additionally, a meta-analysis will only be performed if more than 2 studies are available and explore the same outcomes, and this will be analyzed using RevMan (version 5.4.1). The quality and risk of bias will be assessed following the Cochrane risk of bias tool and Jadad checklist.

**Results:**

The process for searching literature review has already started, and this is conducted independently by 2 reviewers using a predefined eligibility and “participants, intervention, comparator, and outcome” criteria. This systematic review and meta-analysis will not require any direct involvement of patients and the public; thus, no ethical approval was required.

**Conclusions:**

The findings obtained from the proposed study will be presented in scientific seminars, journal clubs, and conferences and published in peer-reviewed journals.

**Trial Registration:**

International Platform of Registered Systematic Review and Meta-analysis Protocols INPLASY202260022; https://inplasy.com/?s=INPLASY202260022

**International Registered Report Identifier (IRRID):**

PRR1-10.2196/42193

## Introduction

Type 2 diabetes (T2D) mellitus is a chronic disease associated with an increased mortality rate [1] due to impaired pathways that regulate homeostatic and inflammatory responses. According to the 2021 International Diabetes Federation report, about 537 million people are estimated to have diabetes worldwide, and this number is expected to reach 783 million in 2045 [2]. T2D is challenging; it affects individual health, while putting a financial burden on the health care system [3,4].

Despite the availability and use of glucose-lowering pharmacological drugs, such as metformin [5], there is a continuous rise in death due to secondary complications associated with T2D globally. The population with T2D reportedly dies from cardiovascular disease more frequently than healthy individuals [6]. Moreover, other patients experience severe side effects from pharmacological drugs [7]. Thus, this is the motivation for exploring different pharmacological and nutraceutical agents to find one with potent antihyperglycemic and hypolipidemic potentials.

Other studies have explored the beneficial impact of natural antioxidants on glucose control, inflammation, and lipid metabolism in metabolic conditions [8-11]. For their distinct antioxidant benefits, many dietary compounds, including vitamin D, are increasingly being taken as supplements. Vitamin D, also called calciferol [12], is a membrane antioxidant and a member of fat-soluble vitamins that alleviates inflammation by inhibiting nuclear factor kappa-beta activity [13,14]. The direct stimulation of pancreatic β cells to release insulin [15], anti-inflammatory effects [16] to reduce chronic inflammation brought on by insulin resistance, and downregulation of elevated parathyroid hormone levels [17], which inhibit insulin secretion [18], are some of the protective mechanisms of vitamin D that have been proposed. In addition to being present in dietary supplements, a small amount can be found in oily fish, red meat, egg yolk, liver, and fortified cereals [19].

A previous meta-analysis confirmed vitamin D’s beneficial effects in regulating blood glucose; however, the improvement was minimal [20]. This effect might be attributed to vitamin D’s unique property in alleviating oxidative stress and inflammation [21]. Although previous quantitative studies have shown that vitamin D supplementation in patients with T2D can reduce inflammation [22,23] and lipid profiles [24], the results are inconsistent, and the markers evaluated are not common. Additionally, the effect of vitamin D supplementation on diabetes-related complications is controversial since most randomized controlled trials (RCTs) conducted used small sample sizes or have used different doses of vitamin D. Consistently, quantitative evidence has not looked at these parameters simultaneously to explore the antioxidant effect of vitamin D in T2D. This led us to propose this systematic review and meta-analysis that will simultaneously investigate the antioxidant benefits of vitamin D in all these parameters in T2D. We aimed to conduct this systematic review and meta-analysis to evaluate the impacts of vitamin D supplementation in T2D and help to further clarify its beneficial impact on inflammation and lipid profile in patients with T2D. This information is essential for understanding the health benefits of this dietary antioxidant and reducing the number of T2D individuals who succumb to cardiovascular disease complications associated with T2D.

## Methods

### Overview

This systematic review and meta-analysis protocol is prepared in accordance with the PRISMA-P (Preferred Reporting Items for Systematic review and Meta-Analysis Protocols) 2015 checklist [25] and the population, intervention, comparison, and outcome (PICO) guideline [26]. The protocol for this systematic review and meta-analysis is registered with the International Platform of Registered Systematic Review and Meta-analysis Protocols (INPLASY202260022) and is reported according to the PRISMA-P (Preferred Reporting Items for Systematic Review and Meta-Analysis Protocols) checklist (Multimedia Appendix 1).

### Purpose of the Study

This proposed study aimed to evaluate the effect of vitamin D on markers of inflammation and lipid profile among adult patients with T2D.

The research question is “Can supplementation with vitamin D improve inflammation and ameliorate dyslipidemia in T2D?” This protocol will subscribe to PICO guidelines [26]. Participants are adult patients with T2D. The intervention will be vitamin D supplementations of any dose, and the comparators are placebo or healthy patients without treatment.

The outcomes are inflammation and dyslipidemia, and the study design includes RCTs.

### Inclusion and Exclusion Criteria

The inclusion and exclusion criteria will be carried out following our PICO guidelines. The review will include RCTs that have focused on adult patients with T2D. There will be no language limitations, and all studies published from inception until December 30, 2022, will be considered. The reviews, books, letters, and animal studies experimenting with diabetes will be excluded. Furthermore, cross-sectional studies, cohort studies, case-control studies, and case reports will all be excluded.

### Search Strategy and Information Sources

Reviewer 1 (RM) and reviewer 2 (KM) will independently conduct a comprehensive literature search through different databases (eg, Embase, Google Scholar, Web of Science, PubMed, and Science Direct). The following keywords (Medical Subject Headings terms), including their synonyms, will be applied in all databases: “Vitamin D,” “calciferol,” “Dihydroxycholecalciferol,” and “type 2 diabetes mellitus.” Furthermore, the evidence will be sought from RCTs published from inception until December 30, 2022, with no restriction regarding publication language. The preliminary search strategy is presented in Table 1 and Table 2.

**Table 1:** Search strategy adapted to indicate preliminary search in a database (PubMed).

Item	MeSH^a^ terms	Search
1	Vitamin D	vitamin D[MeSH Terms]
2	Calciferol	Calciferol[MeSH Terms]
3	1,25-Dihydroxycholecalciferol	1,25-Dihydroxycholecalciferol[MeSH Terms]
4	Type 2 diabetes mellitus	Type 2 diabetes mellitus[MeSH Terms]1
5	1,2, and 3	((Vitamin D[MeSH Terms]) OR (Calciferol[MeSH Terms])) OR (1,25-Dihydroxycholecalciferol[MeSH Terms])
6	4 and 5	(Type 2 diabetes mellitus[MeSH Terms]) AND (((vitamin D[MeSH Terms]) OR (Calciferol[MeSH Terms])) OR (1,25-Dihydroxycholecalciferol[MeSH Terms]))

^a^MeSh: Medical Subject Headings.

**Table 2:** Search strategy on PubMed with filter according to study designs (randomized controlled trials).

Items	MeSH^a^ terms	Search
7	6	(Type 2 diabetes mellitus[MeSH Terms]) AND (((vitamin D[MeSH Terms]) OR (Calciferol[MeSH Terms])) OR (1,25-Dihydroxycholecalciferol[MeSH Terms])). Filters: Randomized Controlled Trial

^a^MeSh: Medical Subject Headings.

### Study Selection

The search will be conducted independently by 2 reviewers (RM and KM) to ensure accuracy and consistency based on the eligibility criteria. Firstly, keywords, titles, and abstracts will be screened, followed by retrieval of the full study. Mendeley desktop reference manager (version 1.19.4) will be used to save studies retrieved through web-based database search or manual reference screening. All studies that meet the inclusion criteria will be subjected to a data collection process and quality assessment. For any discrepancy, the 2 reviewers will conclude by discussing and reevaluating the study in dispute. The Shiny app [27] will be used to create a PRISMA flow diagram showing study selection.

### Data Items and Collection

The data will be extracted following the eligibility criteria. The 2 reviewers RM and KM will conduct data extraction independently and then compare the spreadsheet; in case of disagreement, they will discuss and review the study again. The information sought from each study for extraction will include the author and year of publication, the country where the study was conducted, sample size, participants’ gender and age, form of vitamin D and its dosage, duration of treatment, lipid profiles, and pro-inflammatory markers. Data will be confirmed by contacting investigators if insufficient information has been provided in the published article.

### Risk of Bias

The risk of bias will be assessed using the methods recommended in the Cochrane risk of bias assessment tool [28]; assessment includes 6 domains; selection, attention, reporting, performance, attrition, and other bias. Judgments will be used to determine whether the bias is a high-risk or low-risk bias, is unclear due to insufficient information, or is a potential bias. The Jadad guideline will be used to assess the quality of individual studies [29]. The author (RM) and supervisor (KM) will discuss and resolve any bias and come to a consensus should there be any disagreement.

### Subgroup Analysis

Following initial data analysis, we will evaluate the level of heterogeneity based on *I*^2^ [30] and Q tests. If the *I*^2^ is moderate (*I*^2^≥ 50%), we will explore the source of heterogeneity by performing subgroup analysis [31]. This will be based on the form of vitamin D, its dosage, BMI, study quality, the origin of the study, age, and the duration of the intervention.

### Publication Bias

Publication bias among the included studies will be assessed and visualized graphically through funnel plots [32] if there are more than 10 studies included in the meta-analysis. In case of no publication bias, the funnel plot will form a symmetrical (ie, inverted) funnel. In contrast, if bias is present due to smaller studies without statistically significant effects, the funnel plot will show an asymmetrical shape.

### Sensitivity Analysis

Sensitivity analyses will be performed to evaluate the robustness and stability [33] of our effect size by using one study exclusion approach at a time based on the study with a high risk of bias, small sample size, or the largest sample size.

### Certainty of Evidence

We will evaluate the certainty of our evidence by using the Grading of Recommendations, Assessment, Development, and Evaluations (GRADE) approach [34] using the web-based GRADEprofiler. This approach takes into consideration the risk of bias, imprecision, inconsistency, indirectness, publication bias, and effect size. The evidence will be rated as low, moderate, or high based on 2 independent reviewers’ ratings. The overall findings will be presented in a summary of the findings table.

### Data Analysis

Statistical analysis will be conducted using the Review Manager (RevMan; version 5.4; Cochrane Collaboration). Each study reviewer (RM and KM) will independently extract data as mean, SD, and sample size in each group. Where mean and SD values are not reported, the estimation will be made using a web-based calculator by Hozo et al [35]. The forest plots will be used to determine the pooled estimates and classify the level of heterogeneity based on *I*^2^ and *P* values [36]. The estimated pool will be reported as either mean difference or standardized mean difference and 95% CIs.

Furthermore, a chi-square test (χ^2^) and *I*^2^ will assess the heterogeneity among the included studies [37,38]. An *I*^2^ of 0% to 50% will be considered low to minimal heterogeneity, while an *I*^2^ above 50% will be considered moderate heterogeneity.

Consistently, sources of heterogeneity will be explored through subgroup analysis, and sensitivity analysis will be used to assess the stability of the re-analyzed pool estimates. *P*<.05 will be considered statistically significant. In case meta-analysis cannot be performed due to insufficient data or the number of studies, qualitative synthesis will be performed [39].

### Ethical Considerations

There will be no involvement from patients and the public in this study. This study synthesizes data from studies that were already peer-reviewed and published. Thus, no ethical approval is required.

## Results

The proposed systematic review and meta-analysis is anticipated to be complete and submitted for review by December 30, 2023. Hence, the full results will be discussed in detail following a comprehensive search and data analysis. This systematic review and meta-analysis will not require any direct involvement of patients and the public; thus, no ethical approval is required. The search for literature has already started on MEDLINE using PubMed as a search engine; this is performed independently by 2 reviewers to avoid risk of bias.

## Discussion

In this proposed systematic review and meta-analysis, we will explore the impacts of vitamin D supplementation on markers of inflammation, including tumor necrosis factor-alpha, interleukin-6, and C-reactive protein in patients with T2D. Furthermore, we will evaluate the effect of vitamin D on lipid profile (triglycerides, total cholesterol, low-density lipoprotein, and high-density lipoprotein) in adult patients with T2D. Thus, the findings in this systematic review and meta-analysis will provide insights into the use of this natural antioxidant, vitamin D, as a supplementation in patients with T2D to aid in the prevention of complications associated with T2D. The proposed review will include studies published from inception to December 2022, thus gathering old and current evidence about vitamin D. Additionally, the review will be conducted independently to avoid bias in selection and extraction. One of the major anticipated limitations is methodological heterogeneity due to the use of different doses of vitamin D and duration of intervention, different age groups, and patients with other metabolic conditions in addition to T2D. However, subgroup analysis will be performed according to these confounders to find the source of heterogeneity. This will be through classification of studies based on quality (eg, good or low), doses, age, and duration of intervention. Our discussion will examine the implications of our findings, the limitation of the systematic review, and directions for further research on the use of vitamin D as a supplement in T2D for the amelioration of inflammation and dyslipidemia and prevention of secondary complications.

## Appendix

Multimedia Appendix 1 PRISMA-P checklist. PRISMA-P: Preferred Reporting Items for Systematic Review and Meta-Analysis Protocols.

## Abbreviations

GRADE: Grading of Recommendations, Assessment, Development, and Evaluations
PICO: participants, intervention, comparator, and outcome
PRISMA-P: Preferred Reporting Items for Systematic Review and Meta-Analysis Protocols
PRISMA: Preferred Reporting Items for Systematic Reviews and Meta-Analyses
RCT: randomized controlled trial
T2D: type 2 diabetes

## Data Availability

Information and data involving literature search, data extraction, and data analysis will be made available by the corresponding author upon request following the publication of the manuscript.
